# Effects of scoliosis specific exercise on a 64 y/o woman with degenerative scoliosis

**DOI:** 10.1186/1748-7161-9-S1-O49

**Published:** 2014-12-04

**Authors:** Margaret Gogin, Peter Arndt, Cindy Marti

**Affiliations:** 1 Spinal Dynamics of Wisconsin, Wauwatosa, WI, USA

## Background

Scoliosis specific stabilization exercises are those that are aimed at stabilizing spinal curvatures.

## Aim

The aim of this paper is to assess the efficacy of using corrective exercise on progressive, degenerative curves in an adult population.

## Methods

A 64 y/o, postmenopausal female was seen in the clinic for 10 one hour visits, spaced over 5 months. This patient was significantly concerned regarding the twenty degree progression of her curve over 3 years. Family history is significant for severe spinal stenosis. The patient was educated in safe back mechanics, as well as specific scoliosis stabilization exercises according to Schroth and SEAS. She demonstrated satisfactory quality of exercise. The patient had exercise compliance daily of twenty minutes.

## Results

The patient was seen for annual follow up for the following objective measures: scoliometer angle of trunk rotation, DIERS formetric postural measures, and radiological assessment. We found decreased scoliometer angle of trunk rotation from 8 to 4 degrees, decreased Cobb angle from 35 to 25 degrees, as well as DIERS formetric changes including--improved coronal balance, increased trunk height, increased lumbar lordosis, and decreased surface rotation.

## Conclusion

The results indicate that employing scoliosis specific stabilization exercises may be an effective tool to halt progressive degenerative curves in adult females.

## Consent

Written informed consent was obtained from the patient for publication of this Case report. A copy of the written consent is available for review by the Editor of this journal.

## Competing interests

Spinal Dynamics of Wisconsin has no financial relationship with DIERS.
